# Predicting the Higher Energy Need for Effective Defibrillation Using Machine Learning Based on an Animal Model

**DOI:** 10.3390/jcm14113879

**Published:** 2025-05-30

**Authors:** Ádám Pál-Jakab, Boldizsár Kiss, Bettina Nagy, Ivetta Boldizsár, István Osztheimer, Erika Rózsa Dévényiné, Violetta Kékesi, Zsolt Lóránt, Béla Merkely, Endre Zima

**Affiliations:** 1 Department of Cardiology, Semmelweis University Heart and Vascular Center, 1122 Budapest, Hungary; 2 Data Science, Eötvös Loránd University, 1053 Budapest, Hungary; 3 Innomed Medical—Medical Developing and Manufacturing Inc., 1146 Budapest, Hungary; 4 Institute of Anaesthesiology and Perioperative Patient Care, Semmelweis University, 1096 Budapest, Hungary

**Keywords:** automated external defibrillator (AED), ventricular fibrillation (VF), defibrillation threshold (DFT), machine learning (ML), prediction model

## Abstract

**Background**: Early defibrillation improves outcomes in cardiac arrest, but the optimal defibrillation strategy and energy requirements remain debated. This study investigated whether arterial blood gas (ABG) parameters could predict optimal defibrillation energy requirements for achieving the highest first-shock success rates in an animal model. Our study focused on clinical scenarios where ABG measurements are readily available, such as ventricular tachycardia and ventricular fibrillation storms requiring multiple shock deliveries. **Materials and Methods**: In the experimental setting, ventricular fibrillation was induced by 50 Hz direct current (DC), and the defibrillation threshold (DFT) was determined using a stepwise defibrillation protocol. ABG parameters were measured before each defibrillation attempt, recording partial arterial pressure of carbon dioxide (PaCO_2_) and oxygen (PaO_2_), pH, hematocrit (Hct), sodium (Na^+^), potassium (K^+^), and bicarbonate (HCO_3_^−^) levels. The relationships between ABG parameters and the DFT were analyzed for 15 subjects using classical data analysis techniques and machine learning (ML) algorithms. Multiple ML models were trained and tested to predict the higher energy needed for successful defibrillation based on the ABG parameters. **Results**: Statistically significant differences were found in Hct and Na^+^ levels between the two DFT categories, above 130 Joules (J) and below 40 J (*p* < 0.01). The DFT negatively correlated with PaO_2_ and positively correlated with Hct and Na^+^. However, other ABG parameters did not show significant correlations with DFT. Using ML, we predicted cases requiring higher defibrillation E. Our best-performing model, the Extra Trees Classifier, achieved 83% overall accuracy, with 100% and 67% precision rates for higher and lower DFT categories, respectively. We validated the model using bootstrap resampling and 10-fold cross-validation, confirming consistent performance. We identified Hct, PaCO_2_, and PaO_2_ as significant contributors to model prediction based on the feature importance value. **Conclusions**: Modern data analysis techniques applied to ABG parameters may guide personalized defibrillation energy selection, particularly in controlled clinical environments such as catheterization laboratories and intensive care units where ABG measurements are readily available.

## 1. Introduction

Cardiac arrest (CA) is defined as the sudden and unexpected cessation of heart contraction, accompanied by circulatory collapse, unconsciousness, and loss of breathing [1]. The nontraumatic unexpected death that occurs within one hour of the first onset of symptoms is defined as sudden cardiac death [2].

Ventricular fibrillation (VF) and ventricular tachycardia (VT) are life-threatening arrhythmias causing out-of-hospital CA globally, with a yearly incidence ranging from 67 to 170 per 100,000 people [1,2]. VF accounts for approximately 20% of the total CA cases, and as the most common shockable rhythm, requires immediate cardiopulmonary resuscitation and defibrillation. Early restoration of spontaneous circulation in VF is associated with the highest survival rates [1,3].

Defibrillators are indispensable for terminating pulseless ventricular arrhythmias by electrically resetting the myocardium. Early defibrillation is critical for CA patients with shockable rhythms, significantly improving both immediate and long-term outcomes. Studies have shown that patients who receive early defibrillation have better long-term survival rates, as high as approximately 75%, and an improved quality of life compared to those who do not receive early defibrillation [4,5,6,7]. The efficacy of the first shock is particularly crucial for minimizing no-flow and low-flow times in CA from shockable rhythms [8,9,10,11,12]. The availability of the device, its good technical condition, and the operator’s skill in using the device are also essential factors in the success of the defibrillation [13,14,15].

Clinical defibrillation therapy effectiveness depends on the energy delivered to myocardial cells, determined by preselected voltage and transthoracic impedance. Efficacy is further influenced by external factors such as electrode placement, patch size, and position [16,17], as well as by physical characteristics of biphasic defibrillation waveforms (voltage, timing, and phase tilt) [18]. Impedance—the vector sum of all the forces that act against the electric current flow—is measured by defibrillators using high-frequency signals before chock delivery to precharge capacitors appropriately. During shock delivery, real-time impedance measurements (changes in capacitor terminal voltage) automatically adjust shock duration, allowing the defibrillator device to adapt to patient-specific conditions. Increased impedance affects the delivered energy and typically results in higher defibrillation threshold (DFT) values [19,20].

The 2021 European Resuscitation Council (ERC) guidelines recommend defibrillation energy based on the specific waveform of the device. When the defibrillator’s settings are unknown to the rescuer, the ERC advises using the maximum energy setting for all shocks [21]. However, higher energy levels require longer capacitor charging times, potentially delaying treatment [22]. Therefore, accurately predicting when higher energy is necessary for successful VF or pulseless VT termination would be clinically valuable. Current protocols indicate two minutes of continuous chest compressions following an unsuccessful shock before rhythm reassessment. The exception is in monitored settings (catheterization labs, cardiac surgery operating rooms, intensive care units), where three consecutive shocks may be delivered without intervening compressions.

The DFT is defined as the lowest energy level needed to terminate VF. While DFT measurement is primarily discussed in the implantable cardioverter defibrillator (ICD) literature, where its routine use remains controversial due to battery conservation concerns and contradictory evidence regarding benefits [23,24,25], it represents the most precise method for determining optimal defibrillation energy in experimental settings and specific clinical scenarios. Multiple factors influence DFT values, including metabolic disturbances (acidosis), electrolyte imbalances (hypo-/hyperkalemia), respiratory status (hypoxia), medications (anesthetic agents, antiarrhythmic drugs), physical characteristics (body mass index), technical parameters (shock waveform, transthoracic impedance), and underlying cardiac conditions (congestive heart failure) [20,25,26,27,28].

Despite extensive research on defibrillation efficacy, there remains a gap in understanding how rapidly obtainable physiological parameters might predict optimal energy requirements for successful first-shock defibrillation. Our study addresses this by applying machine learning techniques to arterial blood gas (ABG) parameters in a controlled experimental model. Unlike previous studies that primarily focused on technological aspects or arbitrary energy thresholds, our work employed a systematic protocol to determine precise defibrillation thresholds and correlate them with physiological parameters. By identifying predictive patterns in readily available ABG measurements, our approach offers a pathway toward personalized defibrillation therapy that could improve first-shock success rates, reduce unnecessary high-energy shocks, and ultimately enhance patient outcomes in critical care settings where ventricular arrhythmias are managed.

Our research deliberately focuses on controlled clinical environments such as catheterization laboratories, intensive care units, and emergency departments where ABG measurements are easily accessible. In these settings, arterial lines are often already in place for continuous monitoring and frequent blood sampling during the management of peri-arrest states and malignant arrhythmias. We recognize that in Basic Life Support (BLS) and Advanced Life Support (ALS) settings, particularly in out-of-hospital cardiac arrest scenarios, ABG parameters are typically unavailable, limiting direct application of our approach in those contexts. Our work, therefore, complements, rather than replaces, standardized defibrillation protocols essential for emergency response systems.

## 2. Materials and Methods

### 2.1. Experimental Design and Animal Model

We employed a protocol-based stepwise defibrillation approach in a canine model to investigate relationships between arterial blood gas parameters and defibrillation thresholds (DFT). This methodology allowed us to precisely determine the minimum energy required for successful defibrillation and correlate these values with physiological parameters measured immediately before each attempt. To minimize confounding factors, we implemented a standardized protocol with strict controls throughout the experimental process.

The experimental phase was conducted at Semmelweis University (Budapest, Hungary) in accordance with Good Laboratory Practice regulations. The study was approved by the Ethics Committee of Hungary for Animal Experimentation (date: 22 June 2010, approval number: 22.1/1163/3/2010) and conformed to European Directive 2010/63/EU and the Guide for the Care and Use of Laboratory Animals used by the US National Institutes of Health (NIH Publication No. 86-23, revised 1996).

We used 15 healthy male Beagle dogs (10–11 kg) with normal baseline ABG parameters and no underlying pathologies. Animals were anesthetized with pentobarbital (30 mg/kg IV), administered tramadol (2 mg/kg IV) for analgesia, intubated, and mechanically ventilated. Continuous monitoring was established via invasive blood pressure measurement and ECG. Pediatric defibrillation pads (8 cm diameter, Innomed Medical Zrt., Budapest, Hungary) were placed in a lateral–lateral position on the shaved chest to standardize shock delivery and minimize transthoracic impedance variability. For continuous electrophysiological data recording, a modified prototype defibrillator (legacy motherboard of Innomed Cardio-Aid® 360-B) connected to a personal computer and an Innomed Cardio-Aid® 360-B backup defibrillator (Innomed Medical Zrt., Budapest, Hungary) were utilized.

To ensure measurement consistency, all procedures were performed by a team of experienced professionals following a uniform methodology and timeline. We conducted a comprehensive evaluation of preprocedural, intraoperative, and postoperative procedures to minimize operator-related variability.

### 2.2. Defibrillation Protocol

Biphasic truncated waveform shocks were utilized for all defibrillation attempts. Transthoracic impedance was continuously monitored, with energy adjustments made to compensate for impedance changes from successive shocks, ensuring consistent energy delivery across attempts. Ventricular fibrillation was induced using 50 Hz alternating current for 3 s. After a 10-s stabilization period to allow VF establishment, we initiated our defibrillation protocol, as follows:

1. Initial shock set to 100 J;
2. If successful: Energy decreased by 10 J for subsequent attempts until failure (step-down);
3. If unsuccessful: Energy increased by 10 J per attempt (step-up protocol);
4. The lowest successful energy was recorded as the DFT;
5. After two consecutive failures, a 150 J rescue shock was delivered.

A 3-min stabilization period followed each successful defibrillation to allow hemodynamic parameters to normalize before the next induction. During this period, we continuously monitored vital signs to ensure complete recovery. ABG samples were collected immediately before each VF induction, measuring PaCO_2_, PaO_2_, pH, hematocrit, sodium, potassium, and bicarbonate levels.

**Figure 1:**
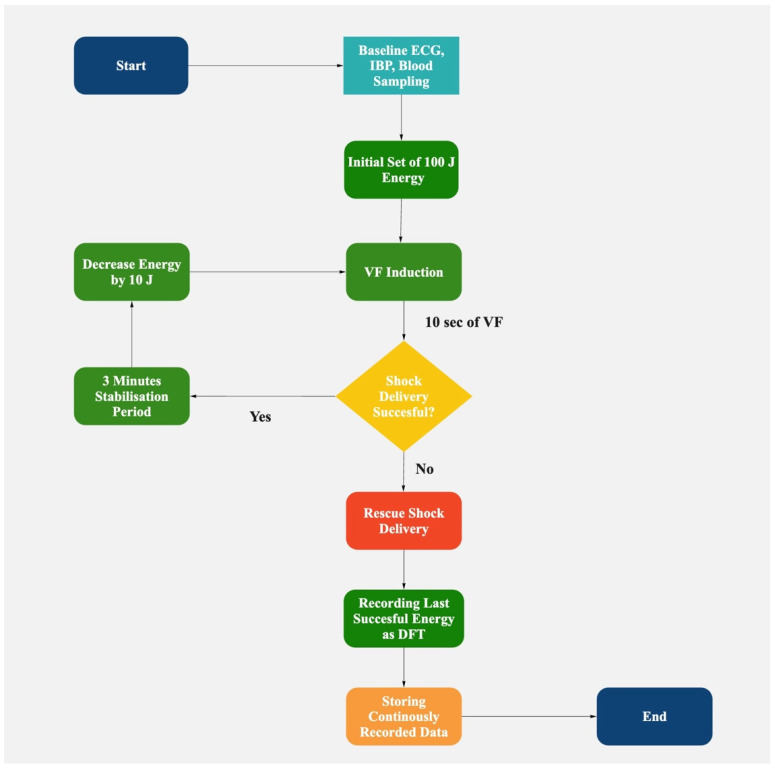
Stepwise defibrillation protocol. The process of the defibrillation threshold (DFT) determination began with baseline measurements, including ECG, invasive blood pressure (IBP), and blood sampling. Following this, the protocol entered a cycle where the defibrillation energy was decreased by 10 J for each subsequent attempt. Ventricular fibrillation (VF) was induced, and after a 10-s period to allow VF stabilization, a shock was delivered at the current energy level. If the shock successfully terminated VF, a 3-min stabilization period was observed to allow hemodynamic parameters to normalize before proceeding to the next attempt at lower energy. This cycle continued with progressively lower energies until a shock failed to terminate VF. At this point, the energy level of the last successful shock was recorded as the DFT and a high-energy rescue shock of 150 J was delivered. If the initial 100 J energy shock did not defibrillate successfully, a step-up protocol was implemented, delivering energy shocks increased by 10 joules for each consecutive unsuccessful attempt.

We planned to analyze the protocol-based serial defibrillation efficacy testing database to determine the cases and predict instances when the defibrillation threshold is higher in an animal model. ABG samples were collected immediately before each defibrillation attempt in the DFT determination process. The DFT was defined as the lowest energy level that successfully terminated VF in our stepwise defibrillation protocol (Figure 1). The DFT values could have only been confirmed retrospectively after a failed shock at a lower energy level was recorded. By correlating ABG parameters with DFT values obtained through our stepwise defibrillation protocol, we aimed to develop a predictive model to identify scenarios where higher initial energy might be beneficial, potentially enhancing the management of malignant/ventricular arrhythmias.

To reduce the impact of known confounding factors on DFT value and enhance our results’ accuracy, we have followed a strict standardized protocol in the experimental phase of the research.

### 2.3. Data Collection and Management

DFT values were determined as the lowest successful defibrillation energy in our protocol. In cases requiring the step-up protocol, the lowest successful energy was still recorded as the DFT. Throughout the experiment, we continuously recorded all shock energies, success/failure outcomes, and corresponding physiological data.

All measurements were initially recorded in separate datasheets, then systematically merged into a unified database containing DFT values and corresponding ABG parameters. This allowed us to assess relationships between ABG values and energy requirements for successful defibrillation.

Data analysis was conducted using Python3 (version 3.10.9., Python Software Foundation, Wilmington, DE, USA), Visual Notebook Mac (version 6.5.2., Project Jupyter, Berkeley, CA, USA) [29], and Visual Studio Code for Mac (version 1.77.2., Microsoft Corporation, Redmond, WA, USA) to build multiple models. For the processes of data analysis and model creation, we used the following Python libraries: imbalanced-learn (v.0.10.1) [30], matplotlib (v. 3.7.1), notebook (v.6.4.8), numpy (v.1.22.4) [31], pandas (v.1.5.3) [32], scikit-learn (v.1.2.2) [33], scipy (v.1.10.1) [34], seaborn (v.0.12.2) [35], and statsmodels (v.0.13.5) [36]. Table 1 presents descriptive statistics of all measured parameters from 113 DFT determinations across 15 animals.

### 2.4. Data Processing and Analysis

#### 2.4.1. Data Exploration and Visualization

We investigated the relationships between laboratory parameters using correlation heatmaps and scatter plots to identify patterns, verify multicollinearity, and assess prediction possibilities. These methods allow significantly faster and more efficient data processing. Furthermore, complex diagrams can also be plotted to enhance the visual interpretation of the results.

Missing values were individually assessed for each variable, with any rows missing DFT values removed from the analysis. Normal distribution of the variables was assessed using the Shapiro–Wilk tests (threshold *p* < 0.05), and groupwise comparisons between DFT groups were performed using repeated measures ANOVA (parametric) and the Kruskal–Wallis test (non-parametric) tests, as appropriate, with a significance level of *p* < 0.05. Outliers were identified through scatter plots and individually assessed due to dataset size constraints.

**Figure 2:**
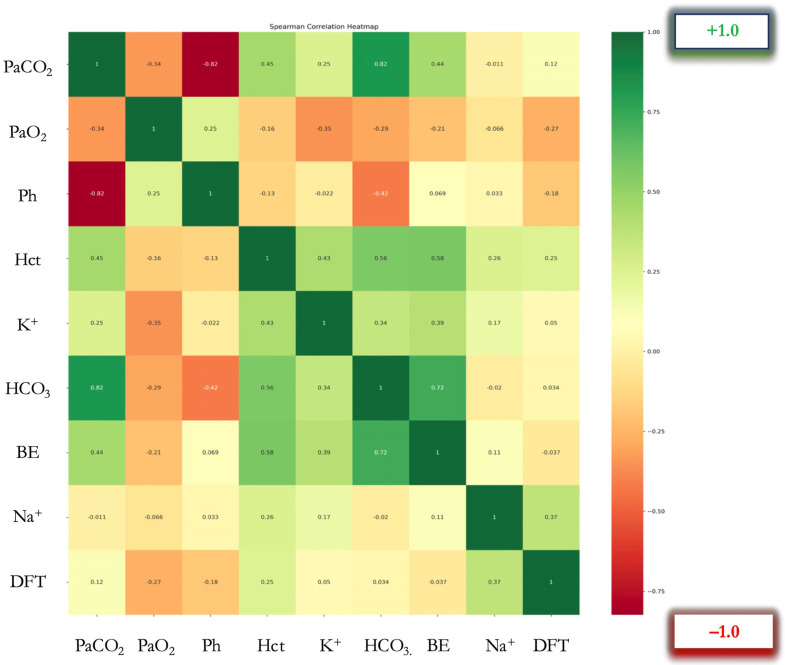
Spearman’s correlation heatmap.

Multiparametric Spearman correlation analysis was performed, and a color-coded correlation heatmap was produced. A dual visual coding was used in this figure: color coding, representing positive and negative correlations, and a second visual element depicting the intensity of the correlation, via the saturation of the color, with darker indicating a strong correlation and lighter indicating a moderate or non-existent correlation (Figure 2). To determine the correlation between data pairs, Spearman’s rank correlation coefficients (ρ) were calculated (Table 2).

Correlation heatmap of arterial blood gas parameters and defibrillation threshold (DFT) (*N* = 113 determinations). Colors represent Spearman’s rank correlation coefficients (ρ): red indicates negative correlation, green indicates positive correlation. Color intensity represents correlation strength as shown in the color mapping bar. Abbreviations: PaCO_2_ (Partial Arterial Pressure of Carbon Dioxide, mmHg), PaO_2_ (Partial Arterial Pressure of Oxygen, mmHg), pH (no units), Hct (Hematocrit, %), K^+^ (Potassium, mmol/L), HCO_3_^−^ (Bicarbonate, mmol/L), BE (Base Excess, mmol/L), Na^+^ (Sodium, mmol/L), DFT (Defibrillation Threshold, Joules)

**Figure 3:**
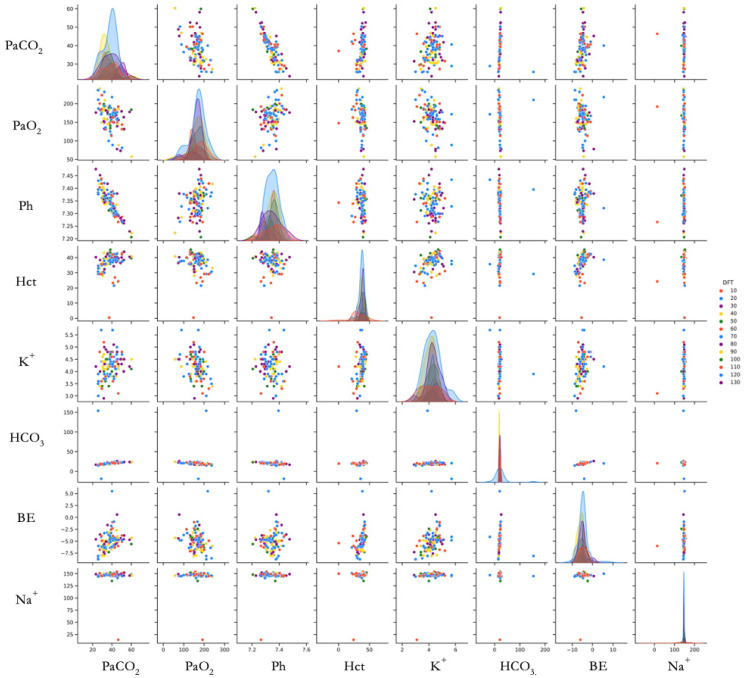
Scatter plot matrix.

To visualize variable relationships comprehensively, we created a scatterplot matrix using the Seaborn library [35]. This matrix displays pairwise relationships between variables, allowing simultaneous evaluation of linear relationships, distribution patterns, and outliers. The Pairgrid function generated a grid colored according to DFT values (Figure 3).

Scatter plot matrix showing relationships between measured parameters and DFT (*N* = 113 determinations from 15 animals). Each point represents a single DFT determination. Color gradient indicates DFT value (J). Diagonal plots show the distribution of each parameter. Abbreviations: PaCO_2_ (Partial Arterial Pressure of Carbon Dioxide, mmHg), PaO_2_ (Partial Arterial Pressure of Oxygen, mmHg), pH (no units), Hct (Hematocrit, %), K^+^ (Potassium, mmol/L), HCO_3_^−^ (Bicarbonate, mmol/L), BE (Base Excess, mmol/L), Na^+^ (Sodium, mmol/L), DFT (Defibrillation Threshold, Joules). DFT values are shown with different colors.

#### 2.4.2. Data Pre-Processing

Following initial exploration, we implemented data pre-processing steps to optimize predictive performance. We assessed multicollinearity using the variance inflation factor (VIF), with values exceeding 5 considered indicative of significant collinearity [37,38]. Variables with high VIF values (pH, Na^+^, K^+^) were individually evaluated, and features with high correlation and high VIF were compared pairwise, with only one retained to enhance model stability.

We performed multiple DFT determinations per animal over the course of the experiment, with appropriate rest periods between measurements to ensure physiological recovery. To account for repeated measures, we employed mixed-effect models in our statistical analysis, treating the animal as a random effect. This approach allowed proper assessment of relationships between ABG parameters and DFT while accounting for within-subject correlations.

Our objective was to develop a clinically relevant predictive model for optimizing defibrillation success. We sorted the database by the magnitude of DFT values, and new subgroups were formed by assigning the highest DFT value to each group member, aiming to predict cases likely requiring higher initial energy for successful defibrillation. The most accurate method proved to be grouping the DFT values into two DFT classes. Since the original data analysis plan proposed running classification algorithms, there was minimal loss of data in the new DFT groupings. Based on the value counts, the best-performing models were those that most precisely predicted higher and lower DFT values based on the input variables.

### 2.5. Machine Learning Model Development

We applied machine learning (ML) techniques to predict DFT values based on ABG parameters. The application of ML in this context aligns with growing recognition of AI’s potential to enhance clinical decision-making in critical care [39,40].

Several classification ML models were trained and evaluated to predict higher DFT values. The models considered include Logistic Regression, Decision Tree, Random Forest (Information Gain), Random Forest (Entropy), Extra Trees Classifier, Support Vector Machines, and XGBoost [33,41,42,43,44,45,46]. To identify the best-performing model, hyperparameter tuning was applied to retrain the models. Hyperparameters in machine learning are external configuration settings that govern the learning process of a model and that can be set prior to training to optimize model performance [47]. At first, models were trained on the original hyperparameter settings; afterward, grid search was used to explore different combinations of hyperparameters. The Extra Trees Classifier emerged as the top-performing model on the validation set.

To ensure robust model fitting and optimal hyperparameter selection, we employed k-fold cross-validation with k set to 5. This technique involved partitioning the training data into k subsets, using one subset for validation while training the model on the remaining k − 1 subsets. The models were precisely fine-tuned to achieve optimal performance by systematically iterating through various hyperparameter configurations. This enabled pinpointing the optimal hyperparameters that would yield the highest model performance, ensuring that our models were well-trained for their intended predictive tasks. The detailed hyperparameter tuning is included in Supplementary Material.

The Extra Trees Classifier emerged as our top-performing model. This algorithm builds multiple decision trees on random feature subsets using a variant of the Random Forest approach, then aggregates their outputs for prediction. Its advantages include increased tree diversity, reduced bias-variance trade-off, resistance to noise and overfitting, computational efficiency, and interpretable feature importance analysis [45]. These characteristics made it particularly suitable for our clinical prediction task with multiple physiological parameters.

**Figure 4:**
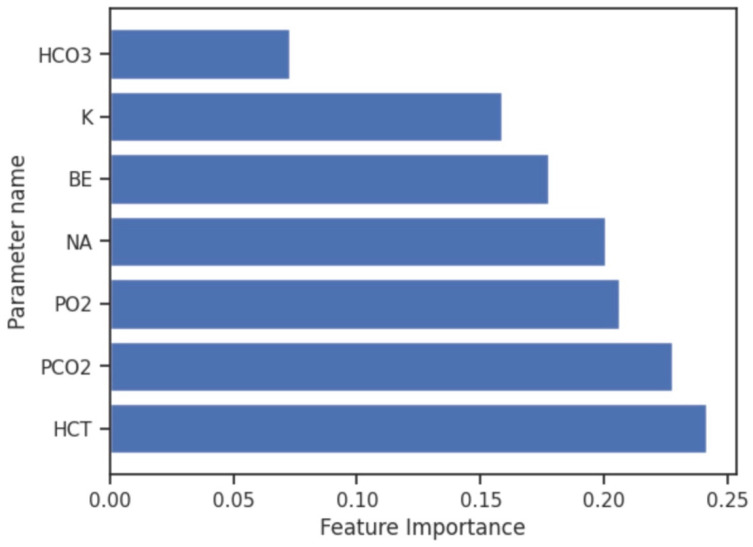
Contribution percentage of the laboratory parameters to the Extra Trees Classifier model based on the feature importance.

To illustrate the accuracy of the best-performing classification model, we plotted a confusion matrix, showing the counts of true positive (TP), false positive (FP), true negative (TN), and false negative (FN) predictions (Figure 4). For a comprehensive assessment of a classification model’s performance, precision and recall are calculated. Precision measures the accuracy of positive predictions, while recall evaluates the model’s ability to identify all actual positive instances. The calculated F1 score provides a single metric to evaluate a model’s accuracy and effectiveness in correctly classifying positive instances while considering both false positives and false negatives.

## 3. Results

In the experimental phase of the research, defibrillations were performed on 15 experimental animals, resulting in 2100 shock deliveries altogether. Based on this data, 113 DFT values were determined using an energy-selective stepwise defibrillation protocol. Less than 1% of missing values per variable were found in the dataset. These missing values were individually assessed and considered to be missing completely at random.

Scatter plot analysis suggested a linear relationship between base excess (BE) and potassium (K^+^), as well as between partial arterial pressure of carbon dioxide (PaCOPaCO_2_) and pH (Figure 3).

Statistical analysis results revealed notable associations between the DFT and specific ABG parameters. The Wilcoxon rank-sum test was employed for pairwise comparisons to evaluate disparities between the two groups categorized by DFT values of 130 J and 40 J. This analysis indicated statistically significant differences in the Hct and Na^+^ levels (*p* ≤ 0.01) between the two groups. No significant differences were observed in the PaCO_2_, PaO_2_, pH, K^+^, HCO_3_^−^, and BE levels between these groups.

A significant negative correlation was observed between PaO_2_ and DFT (ρ = −0.267, *p* = 0.004). Additionally, positive correlations were found between Hct and DFT (ρ = 0.247, *p* = 0.008), as well as between Na^+^ and DFT levels (ρ = 0.372, *p* < 0.001) (Figure 2). Conversely, no significant correlations were detected between other ABG parameters (PaCO_2_, pH, K^+^, HCO_3_^−^, BE, and the DFT).

The VIF analysis revealed moderate collinearity among certain variables. All parameters with VIF values over 5 (pH, Na^+^, and K^+^) were individually addressed. Features with high correlation and high VIF values were compared pairwise and one of each pair was retained in the model training to reduce coefficient inflation and increase the stability of model performance, thus simplifying the dataset for prediction.

The Extra Trees Classifier model proved to be the most effective machine-learning algorithm, demonstrating promising results in identifying patients who may require higher starting defibrillation energy levels. The model achieved an overall prediction accuracy of 83% on the test dataset. Specifically, for patients requiring higher defibrillation energy (the higher DFT value group of above 130 J), the model achieved a precision of 100% and a recall of 75%. For cases with a lower DFT value of 40 J and below, the model achieved a precision of 67% and a recall of 100%. F1-scores were 0.86 for cases necessitating higher energy and 0.80 for cases requiring lower E. These scores provide a balanced measure of precision and recall. Ten-fold cross-validation further assessed the accuracy of the Extra Trees Classifier model, resulting in a 71 ± 0.59% SD accuracy.

Bootstrap resampling with 10,000 replicates provided validation of the model’s performance. The resulting F1 score mean of 0.82 and SD of 0.10 indicates that the classifier consistently attained a relatively high level of accuracy across different bootstrap samples. Similarly, the mean absolute error of 16.19 and SD of 9.03 provide insights into the average deviation between the predicted and actual defibrillation energy requirements.

In our FI analysis, Hct, PaCO_2_, PaO_2_, and Na^+^ emerged as significant contributors in predicting the DFT (Figure 4). Hct and Na^+^ exhibited substantial positive correlations with DFT (ρ = 0.247, *p* = 0.008; ρ = 0.372, *p* < 0.001), as shown in Table 2. In contrast, PaCO_2_ and PaO_2_ ranked as important features in the model but did not display significant correlations with DFT.

Feature importance values from the Extra Trees Classifier model for predicting higher energy need in defibrillation (*N* = 113 determinations). Bars represent the relative importance of each parameter in the model’s decision-making process. Abbreviations: Hct (Hematocrit), PaCO_2_ (Partial Arterial Pressure of Carbon-Dioxide), PaO_2_ (Partial Arterial Pressure of Oxygen), Na^+^ (Sodium), K^+^ (Potassium), BE (Base Excess), and HCO_3_^−^ (Bicarbonate).

**Figure 5:**
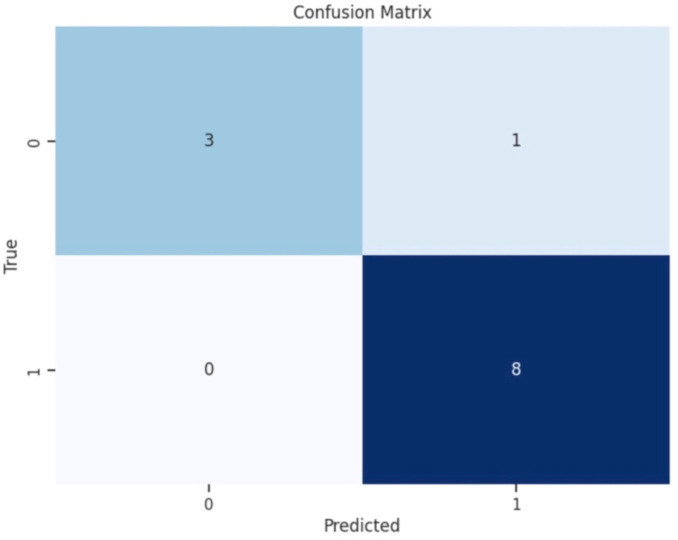
The confusion matrix of the Extra Trees Classifier model.

Our confusion matrix provided a comparison between predicted and actual values. The model correctly predicted 89% of instances of the higher energy class (130 J) as true positives, while incorrectly classifying 11% of cases as a higher energy class when they were actually the lower energy (40 J) class, leading to false positive cases. The model correctly predicted all instances of the lower energy class (40 J) as true negatives and no false negatives (Figure 5).

Confusion matrix of the Extra Trees Classifier model for predicting defibrillation energy requirements (*N* = 113 determinations, 90% training set, 10% test set). True Positive (TP): 3, False Positive (FP): 1, True Negative (TN): 8, False Negative (FN): 0. Overall accuracy: 83%. Label numbers indicate the DFT classes in the following order: Class 0–40 J, Class 1–130 J.

## 4. Discussion

### 4.1. Summary of Key Findings

Our study successfully demonstrated that arterial blood gas parameters can predict defibrillation energy requirements in a controlled experimental model. Using machine learning techniques, specifically the Extra Trees Classifier, we achieved 83% overall accuracy in predicting cases requiring higher defibrillation energy. The most significant predictive parameters were hematocrit, PaCO_2_, and PaO_2_, with hematocrit and sodium showing strong positive correlations with defibrillation threshold values. These findings suggest that rapidly available metabolic and respiratory physiological parameters in controlled clinical settings could inform personalized defibrillation strategies, potentially improving first-shock success rates in specific clinical scenarios.

### 4.2. Comparison with the Existing Literature

Our findings are consistent with the existing literature on factors affecting defibrillation success. Previous studies have shown that electrical factors such as voltage and duration alter transmembrane potential and cardiac tissue refractoriness, impacting wavefront propagation and re-entry circuits [20,48,49]. The relationship between shock characteristics and tissue response is governed by chronaxie and rheobase properties, where rheobase represents the minimum stimulus intensity for successful defibrillation and chronaxie is the duration corresponding to twice the rheobase. Our study extends this understanding by demonstrating that physiological parameters, particularly ABG values, also significantly influence defibrillation energy requirements.

Unlike previous DFT studies that primarily focused on implantable defibrillator contexts with inconsistent protocols [26,27,50,51,52,53], our work employed a standardized stepwise protocol to determine precise defibrillation thresholds. This methodological improvement allowed us to identify previously unreported correlations between ABG parameters and DFT values, particularly the strong positive correlations with hematocrit and sodium levels.

### 4.3. Physiological Mechanisms Underlying Findings

The physiological mechanisms underlying our findings involve complex interactions between ABG parameters and cardiac electrical properties. Hematocrit levels, which showed the strongest positive correlation with DFT (ρ = 0.247, *p* = 0.008), likely influence defibrillation through their effect on blood conductivity and transthoracic impedance. Higher hematocrit ratios increase blood viscosity and may alter electrical conductance, potentially requiring higher energy for successful defibrillation.

Sodium levels also demonstrated a significant positive correlation with DFT (ρ = 0.372, *p* < 0.001). As a primary determinant of cellular membrane potential and spontaneous depolarization, elevated sodium concentrations may stabilize abnormal electrical activity, necessitating higher energy to reset the cardiac rhythm. The negative correlation between PaO_2_ and DFT (ρ = −0.267, *p* = 0.004) suggests that hypoxemia may lower the threshold for successful defibrillation, possibly due to altered cellular metabolism and membrane excitability.

### 4.4. Clinical Implications

The clinical relevance is based on a translational approach to the study, constituting the ground for designing future clinical research and assessing whether ABG or laboratory parameters could forecast increased DFT and, thus, a higher energy need for urgent defibrillation. The clinical applicability of our findings is particularly relevant in controlled medical environments where ABG measurements are readily available and peri-arrest states are commonly managed. In settings such as catheterization laboratories, cardiac intensive care units, and emergency departments with established arterial access, our predictive model could optimize defibrillation strategies for patients experiencing electrical storms or recurrent ventricular arrhythmias.

The ability to predict higher energy requirements based on ABG parameters could improve first-shock success rates, particularly relevant in scenarios where multiple defibrillation attempts are anticipated. This personalized approach complements existing standardized protocols by providing additional decision support in specific clinical contexts where physiological data are already being monitored.

### 4.5. Bridging Theory and Practice: Implementation Considerations

While our research demonstrates theoretical potential and experimental proof for ABG-guided defibrillation energy selection, several practical considerations must be addressed before clinical implementation. The translation from our controlled animal model to human patients requires extensive validation studies accounting for species differences, varying body dimensions, and diverse pathophysiological conditions.

Clinical workflow integration presents another challenge, as predictive algorithms would require seamless integration with existing monitoring systems and defibrillator interfaces. Although ABG measurements are routine in our target settings (ICUs, catheterization labs), effective integration in clinical studies would necessitate healthcare providers’ training on interpreting model outputs while maintaining adherence to established resuscitation protocols.

Additionally, regulatory approval for such predictive systems would necessitate demonstrating not only accuracy but also fail-safe mechanisms to prevent inappropriate energy selection. The system would need to complement, rather than replace, clinical judgment and existing guidelines.

An important consideration is that our approach has limited applicability in Basic Life Support (BLS) and Advanced Life Support (ALS) settings, particularly in out-of-hospital cardiac arrests where ABG measurements are unavailable. In these scenarios, standardized energy protocols remain essential for ensuring consistent timely care. Our model is specifically designed for controlled clinical environments where the benefits of personalization can be realized without compromising the speed and standardization crucial to emergency response.

### 4.6. Limitations of the Study

The database on which this research is based includes data from a series of animal studies. Several limitations should be considered when interpreting our results.

First, our study utilized a relatively small dataset of 2100 shock deliveries and 113 DFT determinations, which may affect the generalizability of our machine learning model. Larger datasets would enhance model robustness and allow for more sophisticated algorithm development.

Second, the stepwise defibrillation protocol used 10 J increments rather than smaller steps, limiting the precision of DFT determination. This constraint, while necessary for practical and ethical reasons in our animal model, may have affected the accuracy of our predictive model.

Third, the use of Beagle dogs, while providing a standardized model, may not fully represent human thoracic anatomy, but their cardiac electrophysiology is as similar to human physiology as that of swine models. Additionally, the controlled experimental environment differs significantly from clinical scenarios where multiple comorbidities and medications may influence defibrillation outcomes.

Fourth, the applicability of our findings is limited to settings where ABG measurements are easily accessible. This excludes most prehospital and emergency scenarios where rapid defibrillation following standardized protocols remains the priority.

### 4.7. Future Directions

Moving forward, validation studies in human subjects are essential to advance this field, particularly in controlled clinical settings where our approach is most applicable. These studies should incorporate larger more diverse patient populations with various cardiac pathologies to ensure the generalizability of findings.

Technological development should simultaneously focus on creating integrated systems that can analyze ABG parameters in real-time and provide energy recommendations without disrupting clinical workflows. This includes developing user-friendly interfaces and fail-safe mechanisms to ensure patient safety while optimizing defibrillation success rates.

Further investigation of additional readily available parameters could enhance predictive accuracy. Exploring electrolytes from point-of-care testing, echocardiographic indices, or continuous cardiac output monitoring might provide valuable complementary data. Combining multiple data sources through advanced machine learning techniques may provide even more personalized defibrillation strategies.

Ultimately, the goal is to develop evidence-based personalized protocols that optimize defibrillation success while maintaining the standardization necessary for emergency medical systems. This balance between personalization and standardization represents the future of precision medicine in resuscitation science, building upon our demonstration that ABG parameters can effectively predict defibrillation energy requirements with 83% accuracy in controlled clinical settings.

## 5. Conclusions

Our research demonstrates that arterial blood gas parameters can effectively predict defibrillation energy requirements in controlled clinical settings, with the Extra Trees Classifier model achieving 83% overall accuracy in identifying cases requiring higher defibrillation energy. Hematocrit, PaCO_2_, and PaO_2_ from BGA emerged as the most significant predictors, establishing a foundation for data-driven personalization of defibrillation therapy in clinical environments where ABG measurements are routinely available.

While our approach has limited applicability in Basic Life Support and Advanced Life Support settings where ABG measurements may be unavailable, it offers valuable decision support in intensive care units, catheterization laboratories, and emergency departments with established arterial access. These findings complement existing standardized protocols by enabling personalized energy selection in controlled environments, representing a meaningful step toward precision medicine in resuscitation science that requires future clinical validation in human subjects.

## Figures and Tables

**Table 1:** Descriptive statistics of measured parameters (*N* = 113 DFT determinations from 15 animals). Values are presented as median, 25th and 75th percentiles, and standard deviation (SD). The Shapiro–Wilk test was used to assess the normality of distribution. Abbreviations: BE (Base Excess), DFT (Defibrillation Threshold), HCO_3_^−^ (Bicarbonate), Hct (Hematocrit), K^+^ (Potassium), Na^+^ (Sodium), PaCO_2_ (Partial Arterial Pressure of Carbon Dioxide), pH, and PaO_2_ (Partial Arterial Pressure of Oxygen).

	Median	25th Percentiles	75th Percentile	SD
BE (mEq/L)	−4.90	−6.20	−4.30	1.93
DFT (J)	40.00	20.00	60.00	29.62
HCO_3_^−^ (mEq/L)	20.30	18.10	21.80	13.33
Hct (%)	38.40	35.70	40.20	5.66
K^+^ (mmol/L)	4.20	3.90	4.50	0.49
Na^+^ (mEq/L)	147.00	146.00	149.00	12.77
PaCO_2_ (mmHg)	38.20	32.10	43.00	7.47
PaO_2_ (mmHg)	167.20	148.40	183.70	32.57
pH	7.35	7.31	7.38	0.05

**Table 2:** Spearman’s correlation coefficients.

Parameter Name	Spearman Correlation with DFT	*p*-Value
BE	−0.0371	0.696
HCO_3_^−^	0.0339	0.722
Hct	0.2475	**0.008**
K^+^	0.0583	0.539
Na^+^	0.3728	**<0.001**
PaCO_2_	0.1246	0.189
pH	−0.2117	**0.024**
PaO_2_	−0.2671	**0.004**

Spearman’s rank correlation coefficients (ρ) between measured parameters and Defibrillation Threshold (DFT) (*N* = 113 determinations). Statistically significant correlations (*p* < 0.05) are in bold. Abbreviations: BE (Base Excess), DFT (Defibrillation Threshold), HCO_3_^−^ (Bicarbonate), Hct (Hematocrit), K^+^ (Potassium), Na^+^ (Sodium), PaCO_2_ (Partial Arterial Pressure of Carbon Dioxide), pH, PaO_2_ (Partial Arterial Pressure of Oxygen).

## Data Availability

The datasets analyzed during the current study are not publicly available due to ethical restrictions and the sensitive nature of the experimental data involving animal subjects. However, datasets may be made available from the corresponding author upon reasonable request. Requests to access the datasets should be directed to Endre Zima, zima.endre.istvan@semmelweis.hu.

## Supplementary Materials

The following supporting information can be downloaded at https://www.mdpi.com/article/10.3390/jcm14113879/s1.

## Abbreviations

The following abbreviations are used in this manuscript:

ABG	Arterial blood gas
ACC	American College of Cardiology
AED	Automated external defibrillator
AHA	American Heart Association
AI	Artificial intelligence
BE	Base excess
CA	Cardiac arrest
CLI	Heroku Command Line Interface
DC	Direct current
DFT	Defibrillation threshold
ERC	European Resuscitation Council
F1 score	F1 measure
FI	Feature importance
FN	False negative
FP	False positive
GLP	Good Laboratory Practice
Hct	Hematocrit
HCO_3_^−^	Bicarbonate
Hz	Hertz
ICD	Implantable cardioverter defibrillator
J	Joule
K^+^	Potassium
ML	Machine learning
Na^+^	Sodium
PaCO_2_	Partial arterial pressure of carbon dioxide
PaO_2_	Partial arterial pressure of oxygen
RF	Random Forest
ρ	Spearman’s rank correlation coefficient
Std.	Standard
Std. Dev.	Standard deviation
TN	True negative
TP	True positive
VIF	Variance inflation factor
VF	Ventricular fibrillation
VT	Ventricular tachycardia
X_train	Training independent variables of the dataset
y_train	Training dependent variables of the dataset
X_test	Testing variables of the dataset
y_test	Testing variables of the dataset
