# Supplementary material for: Predicting the Higher Energy Need for Effective Defibrillation Using Machine Learning Based on an Animal Model
Source: J Clin Med. 2025 May 30;14(11):3879. doi: 10.3390/jcm14113879 (PMC12156191; doi:10.3390/jcm14113879)
Supplement: Supplementary file 1 [file jcm-14-03879-s001.zip › jcm-3618097-supplementary.pdf]

Supplementary

Detailed Experimental Procedures

1. Animal Model and Preparation

Male Beagle dogs (n=15, weight 10-11 kg) were used in this study.

2. Anesthesia and Surgical Procedure

Anaesthesia was induced with pentobarbital (30 mg/kg IV) and maintained with continuous infusion (8-12 mg/kg/h) titrated to effect. Tramadol (2 mg/kg IV) was administered for analgesia. Throughout the experiment, after intubation, animals were mechanically ventilated with Versamed iVent 201ventilator (GE HealthCare Technologies, Inc., Chicago, Illinois, United States).

Under sterile conditions, a conventional implantable cardioverter defibrillator shock lead (ICD lead, Kentrox RV 65, Biotronik, Berlin, Germany) was inserted via the right jugular vein and advanced under fluoroscopic guidance (Philips BW Pulsera X-ray fluoroscopy device, Philips Amsterdam, Netherlands) to the right ventricular apex. TMS1000 programmer (Biotronik, Berlin, Germany) was connected to the ICD lead and was used for electrophysiology stimulation and VF induction. The left femoral artery was catheterized for continuous blood pressure monitoring and sampling.

3. Monitoring and Equipment

Hemodynamic measurements, including invasive arterial blood pressure, heart rate, and oxygen saturation, were recorded at 5-minute intervals. Continuous IBP, ECG monitoring and recording was performed with Hemosys module and software (Experimetria Ltd., Budapest, Hungary) on a desktop computer. Additional continuous ECG monitoring was established via defibrillation pads connected to Innomed Cardio-Aid® 360-B (Innomed Medical Zrt., Budapest, Hungary).

4. Defibrillation Equipment

Pediatric defibrillation pads (8 cm diameter, Innomed Medical Zrt., Budapest, Hungary) were placed in a lateral-lateral position on the shaved chest to standardize shock delivery and establish a hairless transthoracic impedance. Aiming continuous recording of electophysiologic data derived via the patches standard defibrillator hardware connected to PC („prototype" or modified defibrillator) and an Innomed Cardio-Aid® 360-B backup defibrillator (Innomed Medical Zrt., Budapest, Hungary) were set up. The defibrillators deliver biphasic truncated exponential (BTE) waveform shocks with settings from 2 J to 360 J. The devices incorporate impedance compensation technology, ensuring consistent energy delivery across all attempts.

5. Experimental Protocol

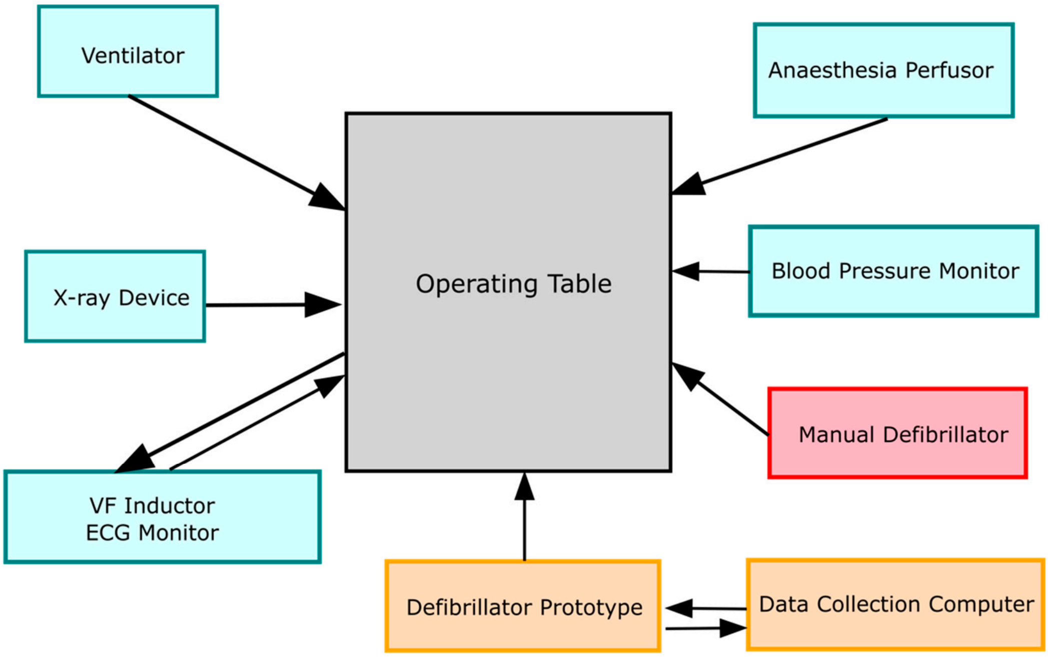

Figure S1. Experimental Setup for the Stepwise Defibrillation Protocol

The figure shows the arrangement of medical equipment around the operating table, including a ventilator, X-ray device, anaesthesia machine, blood pressure monitor, defibrillators, and data collection systems.

5.1. VF Induction and Defibrillation Procedure

We utilized a step-down protocol for DFT determination, starting at 100 J and decreasing by 10 J after each successful defibrillation. VF was induced with 50 Hz stimulation via the ICD lead and TMS1000, and then charging and shock delivery was started after 10 seconds of VF duration. After a failed defibrillation attempt, a 150 J rescue shock was delivered. If the initial 100 J shock failed, we implemented a step-up protocol, increasing energy by 10 J per attempt.

5.2. Stabilization Period

Each successful defibrillation was followed by a 3-minute stabilization period with continuous hemodynamic monitoring. If values did not return to within 10 % of baseline by the end of this period, stabilization time was extended until stability was achieved.

5.3. Blood Sampling and Analysis

Arterial blood samples (1 mL) were drawn immediately before each DFT determination after withdrawing 2 mL to ensure representative sampling. Samples were analyzed within 1 minute using a Roche point-of-care blood gas analyzer.

5.4. Data Acquisition and Management

The defibrillator stored ECG curves and capacitor terminal voltage at 500Hz. Physiological data were continuously recorded using a custom acquisition system via the „prototype" defibrillator (Innomed Medical Zrt.). For each stepwise defibrillation cycle, energy delivered, success/failure, and corresponding baseline ABG values were manually entered into a custom-designed Excel spreadsheet in real time.

Post-experiment data from the Hemosys, Innomed database and Excel were merged, verified for accuracy, and exported as a CSV file for further offline analysis.

5.5. Study Termination

After the experiment, animals were euthanized under deep anaesthesia using Euthasol 40% (400 mg/mL pentobarbital sodium and 50 mg/mL phenytoin sodium) at a dose of 1 mL/4.5 kg IV, followed by 40 mL of 40% potassium chloride in Ringer's solution.

6. Legal Considerations

All procedures were performed in a dedicated large animal laboratory at Semmelweis University, Budapest, Hungary, in accordance with the Ethics Committee of Hungary for Animal Experimentation (22.1/1161/3/2010), European Directive 2010/63/EU, and the Guide for the Care and Use of Laboratory Animals (NIH Publication No. 86-23, revised 1996).
